# Novel Solvent-free Perovskite Deposition in Fabrication of Normal and Inverted Architectures of Perovskite Solar Cells

**DOI:** 10.1038/srep33649

**Published:** 2016-09-19

**Authors:** Bahram Abdollahi Nejand, Saba Gharibzadeh, Vahid Ahmadi, H. Reza Shahverdi

**Affiliations:** 1 Nanomaterials Group, Dept. of Materials Engineering, Tarbiat Modares University, Tehran, Iran; 2 Department of Physics, Tarbiat Modares University, Tehran, Iran; 3 School of Electrical and Computer Engineering, Tarbiat Modares University, Tehran, Iran

## Abstract

We introduced a new approach to deposit perovskite layer with no need for dissolving perovskite precursors. Deposition of Solution-free perovskite (SFP) layer is a key method for deposition of perovskite layer on the hole or electron transport layers that are strongly sensitive to perovskite precursors. Using deposition of SFP layer in the perovskite solar cells would extend possibility of using many electron and hole transport materials in both normal and invert architectures of perovskite solar cells. In the present work, we synthesized crystalline perovskite powder followed by successful deposition on TiO_2_ and cuprous iodide as the non-sensitve and sensitive charge transport layers to PbI_2_ and CH_3_NH_3_I solution in DMF. The post compressing step enhanced the efficiency of the devices by increasing the interface area between perovskite and charge transport layers. The 9.07% and 7.71% cell efficiencies of the device prepared by SFP layer was achieved in respective normal (using TiO_2_ as a deposition substrate) and inverted structure (using CuI as deposition substrate) of perovskite solar cell. This method can be efficient in large-scale and low cost fabrication of new generation perovskite solar cells.

Organometal halide perovskite solar cells attracted much attention within the last five years because of their interesting photovoltaic properties. Many reports introduced numerous advantages of organometal halide perovskite materials such as broad range of light absorption, covering the visible to near infrared spectrum with high extinction coefficient (~10^4 ^cm^−1^ at 550 nm), and long diffusion length around 1 μm^1^,^2^,^3^. To date, the certified power conversion efficiency (PCE) of the perovskite solar cells is 20.1^4^. A variety of device architectures and perovskite deposition methods were introduced to enhance the perovskite solar cells properties. Deposition of perovskite layer is among the most important steps to reach high-efficiency solar cells. A large number of attempts have been conducted to reach a uniform and modified perovskite layer to decrease the carriers recombination and lengthy electrons and holes life-time^5^,^6^,^7^,^8^,^9^. Generally, deposition of perovskite layer is classified into two one-step^10^,^11^ and two-step^12^,^13^ chemical and physical sequential methods^14^,^15^. As reported, deposition methods of perovskite layers result in various morphologies and thicknesses that strongly affect the cell performance and parameters. It was declared that formation of pinhole-free perovskite layers considerably improves the cell parameters, especially the open circuit voltage and fill factor, because of high recombination rate in the interface of electron and hole transport materials^5^,^8^,^15^. Hence, many attempts were undertaken to reach pinhole-free perovskite layers including solution engineering^16^,^17^,^18^,^19^, vapor assisted solution process^8^, two-source vapor deposition^15^,^20^,^21^,^22^, solvent-solvent extraction^7^, blow-drying process^23^,^24^, and a solid state chemistry^25^. Since deposition of perovskite layers by vacuum technique involves high production costs, deposition of perovskite layers by chemical routes attracted much attention because of the ease of fabrication and low costs involved. In this regard, spin coating is considered as the most common deposition method for perovskite layers. However, despite resulting in thin and uniform layers, this method would not be a good candidate for scale-up due to its limitations in large area deposition. As discussed, except physical deposition of perovskite that requires high vacuum condition, almost all reported chemical methods in deposition of perovskite layers including one- and two-step approaches are conducted by dissolving the precursors of metal halides and/or methylammonium halides to form the perovskite layer. In fabrication of normal and invert perovskite solar cells by sensitive electron transfer materials (ETMs) or hole transfer materials (HTMs) such as cuprous iodide, there are some limitations resulting in corrosion during deposition of perovskite on the carrier extraction layers. Hence, removal of the solution base methods would receive much attention in fabrication of sensitive based HTMs and ETMs.

In this work, we introduced a new perovskite deposition approach that is quite appropriate and noncorrosive on sensitive HTM and ETM layers. Deposition of perovskite layer was conducted in solar cell by spray coating of the as prepared perovskite powder suspension on TiO_2_ compact layer followed by hot compressing the spray-coated perovskite. To investigate the corrosive impact of reported method on deposition of perovskite layer, the perovskite powder suspension was sprayed and compressed on the cuprous iodide layer to fabricate the FTO/CuI/SFP/PCBM/Al device. Deposition of the SFP would be suitable for spray coating, spin coating, blade coating, and printing. According to the fact that spray coating is a potential deposition method for large area and continuous deposition, we fabricated the perovskite solar cell devices by spray coating of SFP in various spray coating passes for reaching the optimum surface coverage of perovskite layer.

## Results and Discussion

### Photo-charge Transport and Device Preparation

**Figure 1:**
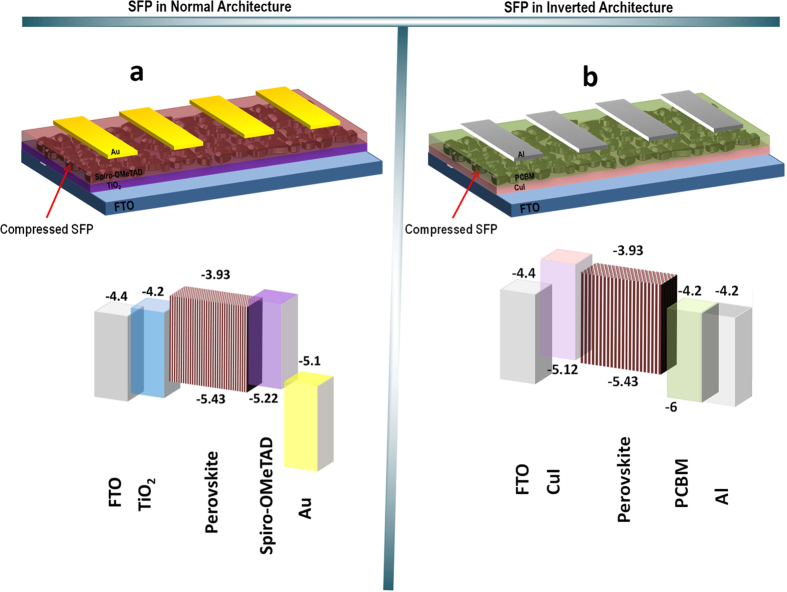
Schematic of device architectures and energy band diagrams of FTO/TiO_2_/SFP/spiro-OMeTAD/Au (**a**) and FTO/CuI/SFP/PCBM/Al (**b**).

Eliminating the need for dissolving perovskite precursors in an appropriate solvent, which is commonly water absorber even at the low moisture ambient atmosphere, would attract much attention in fabrication of perovskite solar cells. Besides, reaching a uniform perovskite layer and a good contact between perovskite and ETM or HTM layers would indicate the great photovoltaic properties in the perovskite solar cells. Hence, these two parameters should be considered in deposition of SFP layer. Figure 1a shows the schematic and band diagram of the prepared device in the normal structure of FTO/TiO_2_/SFP/spiro-OMeTAD/Au. As shown in band diagram of this device, in the normal architecture, the generated electrons are transferred into TiO_2_ compact layer and the generated holes are extracted by spiro-OMeTAD as a HTM (Fig. 1a), while in the inverted architecture of FTO/CuI/SFP/PCBM/Al, the generated electrons are extracted by PCBM and the generated holes are extracted by CuI (Fig. 1b).

In production of perovskite particles, since the sensitivity of perovskite to water molecules and heat is high, the perovskite particles prepared from gas transformation of PbI_2_ particles by methylammonium iodide at 150 °C, were ball-milled in the anhydrous 2-propanol containing a slight amount of methylammonium iodide (5 mg/mL) to protect the perovskite particles from degradation during the ball milling. During the ball milling, simultaneous to grinding the perovskite particles to finer particles, the probable remained lead iodide content in the gross perovskite core is transferred into the perovskite structure. Thus, the total conversion of lead iodide to perovskite structure is completed, while the perovskite particles are ground to smaller particles for better surface coverage and reaching lower thickness during the spray coating.

### SFP Preparation and Deposition

**Figure 2:**
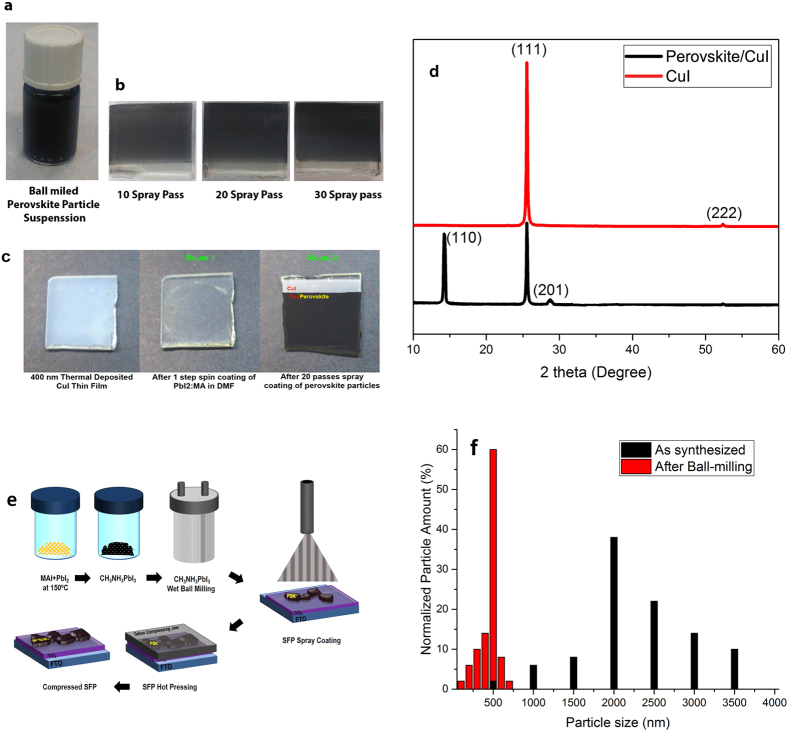
Prepared SFP suspension (**a**), deposited SFP on the TiO_2_ at different spray passes (**b**), conventional one step spin coating of perovskite (route 1) and deposited SFP on CuI at spray passes of 20 (route 2) (**c**), and XRD patterns of bare CuI and CuI/SFP layers (**d**), schematic of synthesizing and deposition process of SFP layer (**e**), and size distribution of the perovskite particles before and after ball milling for 6 hours at 250 rpm monitored by DLS with the number averaged (**f**).

The optimum grain size distribution was obtained through the preparation of device with various grain size distributions. The various perovskite particles distribution reached in different ball milling times. Several spray passes were performed to reach the optimum device performance. As shown in Fig. 2a, the prepared perovskite particle suspension shows long-term stability for frequent spray coating. Besides, the spray-coated layers in various spray passes of 10, 20, and 30 present almost good surface coating quality and better surface coverage by increasing the spray passes (Fig. 2b). As previously reported for two-step deposition microstructure enhancement^26^, hot pressing as a post-treatment process increases the contact area of perovskite and TiO_2_ layer and prevents reaching the HTM to ETM layer. Accordingly, the hot pressing was performed in the present work for enhancing the spray coated SFP. In this technique, as-sprayed perovskite particles are compressed by the smooth Teflon sheets, leading to regulating the surface uniformity and squishing the perovskite particles on the TiO_2_ substrate by a diffusion mechanism of atoms under hot pressing. This two-step deposition mechanism avoids the need of dissolving the perovskite precursors in the strong solvent that also considerably affect some substrates such as CuI and Cu_2_O.

Figure 2c shows deterioration of the CuI sublayer by spin coating of 1:1 PbI_2_:CH_3_NH_3_I solution in DMF (route 1), through which cuprous iodide shows notable corrosion sensitivity, while deposition of SFP on the CuI thin film by spray coating does not affect the CuI microstructure or forming other kinds of interphase structures (Fig. 2d). Synthesizing and deposition of SFP layer in the present work is schematically shown in Fig. 2e. The Dynamic light scattering (DLS) measurements were performed to determine the particle size distribution of perovskite particles before and after ball milling for 6 hours. As shown in Fig. 2f, as transformed perovskite particles (in the transformation of PbI_2_ particles to perovskite particles) has a larger grain sizes in the range of 500 to 3500 nm, while by ball milling the perovskite powder, a narrower size distribution range and smaller grain sizes in the range of 100 to 700 nm was achieved.

### Sprayed and Hot-pressed SFP Morphology

**Figure 3:**
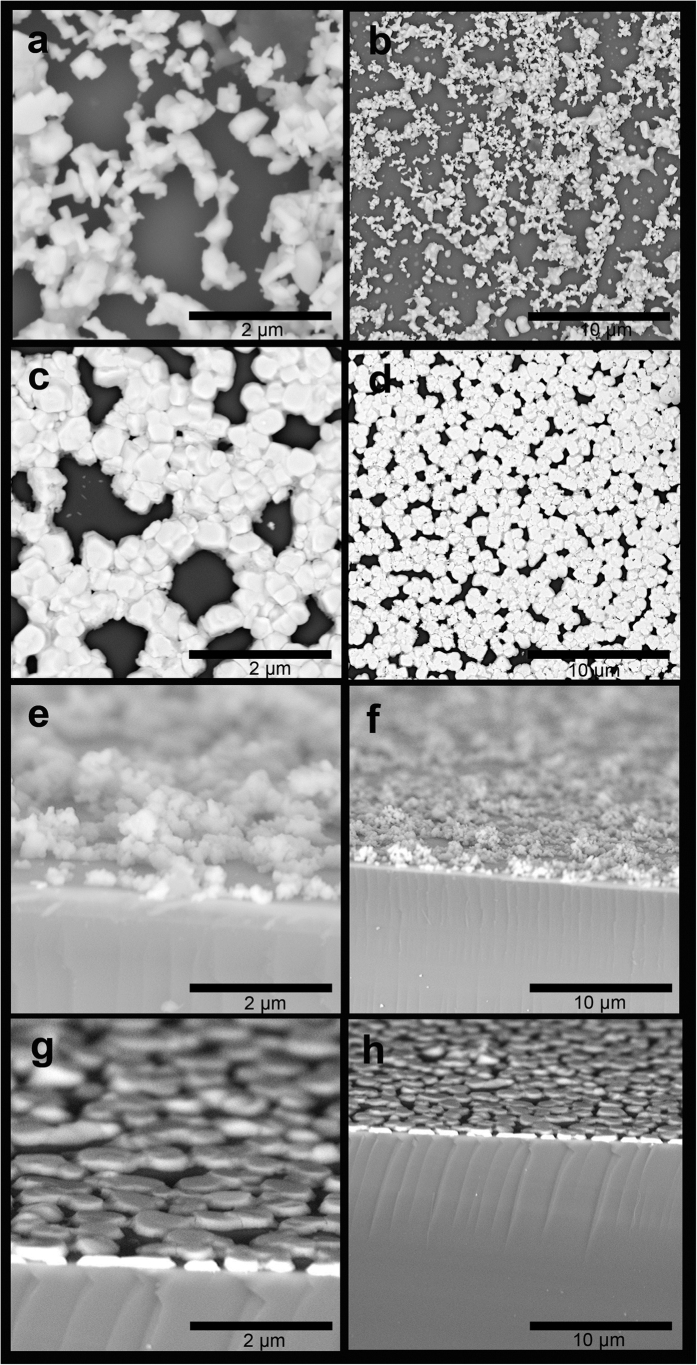
Top and cross sectional FE-SEM images of SFP layer by 10 spray passes (**a**,**b**,**e**,**f**) and hot compressed SFP layer (**c**,**d**,**g**,**h**).

As described, well surface coverage and good contact between perovskite and HTM or ETM layers are very crucial in fabrication of SFP layer. Therefore, in the present work, we utilized hot pressing to reach a better contact of perovskite on the sublayers of TiO_2_ and CuI by reaching an appropriate surface coverage through the optimum number of spray passes. Figure 3 illustrates top and cross section of 10 passes spray coated perovskite suspensions on the substrate followed by compression the SFP layer.

**Figure 4:**
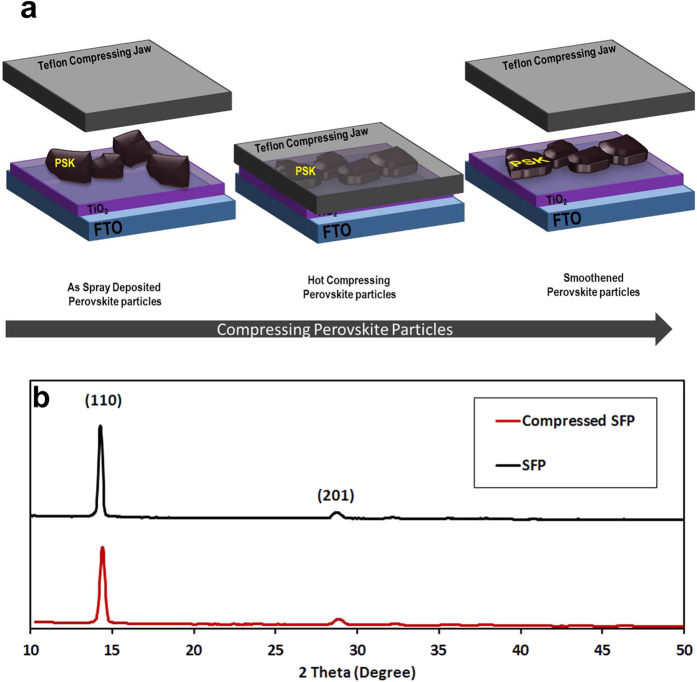
Schematic compression mechanism of SFP by a smooth Teflon compressing jaw (**a**) and XRD patterns of SFP before and after compressing (**b**).

As shown in Fig. 3a,b, as-deposited perovskite particles on the 100 °C substrate do not completely cover the substrate in 10 passes deposition. The 10 pass spray coating of perovskite was chosen to study the perovskite particles landing (Fig. 3a,b,e,f) and compressing mechanism (Fig. 3c,d,g,h) on the substrate through the spray coating followed by hot pressing. As shown in Fig. 3a,b,e,f, as-deposited perovskite layer by perovskite suspension through the spray coating does not cover the surface efficiently due to insufficient spray coating steps and irregular landing of perovskite particles (Figure S1), while a one-step compressing provides perovskite particles with better contacting with substrate (Figs 3c,d,g,h and 4a and Figure S1). According to the fact that perovskite structure may decompose to lead iodide and methylammonium iodide gas in high temperature, we conducted XRD analysis to trace the perovskite microstructure stability in the present hot pressing. As shown in Fig. 4b, and Figure S2, no decomposition of perovskite to PbI_2_ structure was observed during the compressing step.

### Photovoltaic Performance in Normal Architecture

**Figure 5:**
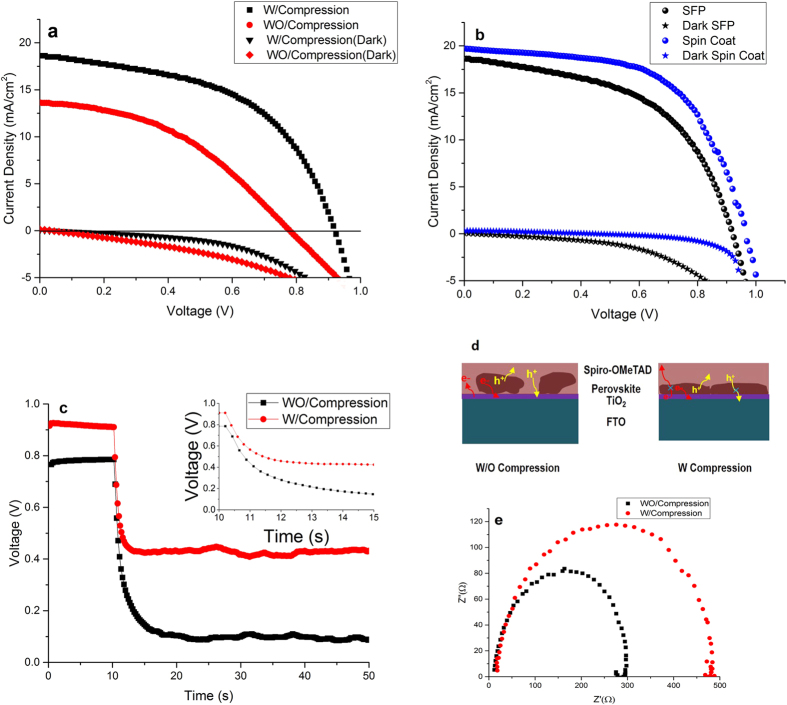
J-V curves measured at AM1.5G solar illumination for devices prepared with normal architecture of FTO/TiO_2_/SFP/spiro-OMeTAD/Au (**a**) and inverted architecture of FTO/CuI/SFP/PCBM/Al (**b**); the photovoltage decay analysis for device structure of FTO/TiO_2_/SFP/spiro-OMeTAD/Au (**c**); schematic of charge transferring and recombination states in SFP based solar cells (**d**), and Nyquist plots of EIS measurements under dark condition with a 0.8 V bias voltage (**e**).

SFP deposition method in preparation of perovskite solar cells was studied by preparation of normal and inverted structures of FTO/TiO_2_/SFP/spiro-OMeTAD/Au and FTO/CuI/SFP/PCBM/Al, respectively. The photovoltaic performance of normal and inverted devices prepared by with and without hot pressing of spray coated perovskite particles was studied in this work. As shown in Fig. 5 and Table 1, the perovskite solar cell prepared by as-deposited perovskite particles through the 20 step spray passes shows PCE of 4.46%, whereas by compressing the perovskite particles by a Teflon jaw, the PCE of device reached 9.06%.

SEM microstructure study for compressing the perovskite particles shows a better connection between the perovskite particles and the substrate. In addition, compressing the perovskite particles unifies the perovskite layer to reach a smoother surface. Hence, enhancement in better interface connection between perovskite particles and electron transport layer would improve the carrier transferring and lower charge recombination in the device, which was shown in the J-V study of the device under dark condition (Fig. 5a). The lower recombination of carriers in the compressed perovskite layer can be attributed to the decline of recombination between spiro-OMeTAD and TiO_2_ because of decreasing the contacting surface of spiro-OMeTAD and TiO_2_^27^ and also the recombination of generated carriers in the perovskite grains through decreasing the thickness of perovskite particles and grains^28^. As shown in the Fig. 5b, the prepared perovskite solar cells by present method still show lower V_oc_ and FF than prepared device by spin coating of perovskite layer, probably because of higher recombination in the TiO_2_/SFP and TiO_2_/spiro-OMeTAD interfaces. It is noteworthy that the perovskite thickness in both devices was almost the same. In addition, the lower current density of prepared device by SFP may be justified by higher resistance in the interface of perovskite and TiO_2_ layer. The photovoltage decay study of the device confirms the higher charge lifetime in the compressed perovskite solar cells (Fig. 5c). As shown in Fig. 5d, the transferred electron and holes, show more possibility of recombine in the interface of spiro-OMeTAD and TiO_2_ layer, while by compressing the deposited perovskite particles on the TiO_2_ layer, because of better surface coverage by squished perovskite particles, there would be a lower possibility in recombination of transferred carriers. To study the adhesion effect, in addition to V_oc_ decay and dark J-V analysis, we added the EIS analysis. As shown in the Fig. 5e, the as-deposited SFP layer by spray coating shows weaker carrier recombination resistance compare to compressed SFP layer. Compression the SFP layer provides higher charge recombination resistance due to better interface contact between SFP particles and TiO_2_ substrates and fewer pinholes in the perovskite layer.

**Figure 6:**
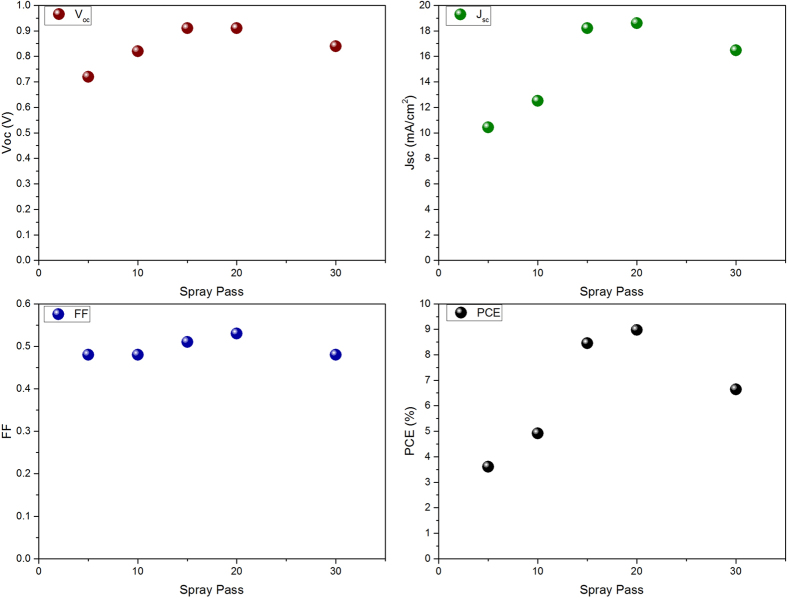
Spray coating passes dependence of SFP layer on device performance parameters.

Figure 6 shows the photovoltaic parameters of perovskite solar cells prepared by various spray coating passes of perovskite solution. As shown in the figure, by increasing the spray coating passes followed by hot compressing up to 20 passes, the current density considerably increases. This behavior can be described by the increase in perovskite content and the better contacting between perovskite particles and TiO_2_ compact layer. Moreover, the open circuit voltage and fill factor of the prepared device is improved because of better surface coverage by compressed perovskite particles by which the recombination sites in the interface of spiro-OMeTAD and TiO_2_ are reduced. At the higher spray passes, thicker perovskite layers are reached through which due to long diffusion path, higher recombination possibility in the perovskite layer is observed. The statistic data of the prepared devices is shown in Figure S3. As shown in the figure, the prepared devices shows good reproducibility. The present reproducibility of devices would attract much attention in scaling up the perovskite solar cells fabrication.

### Optimized Hot-pressed SFP Morphology

**Figure 7:**
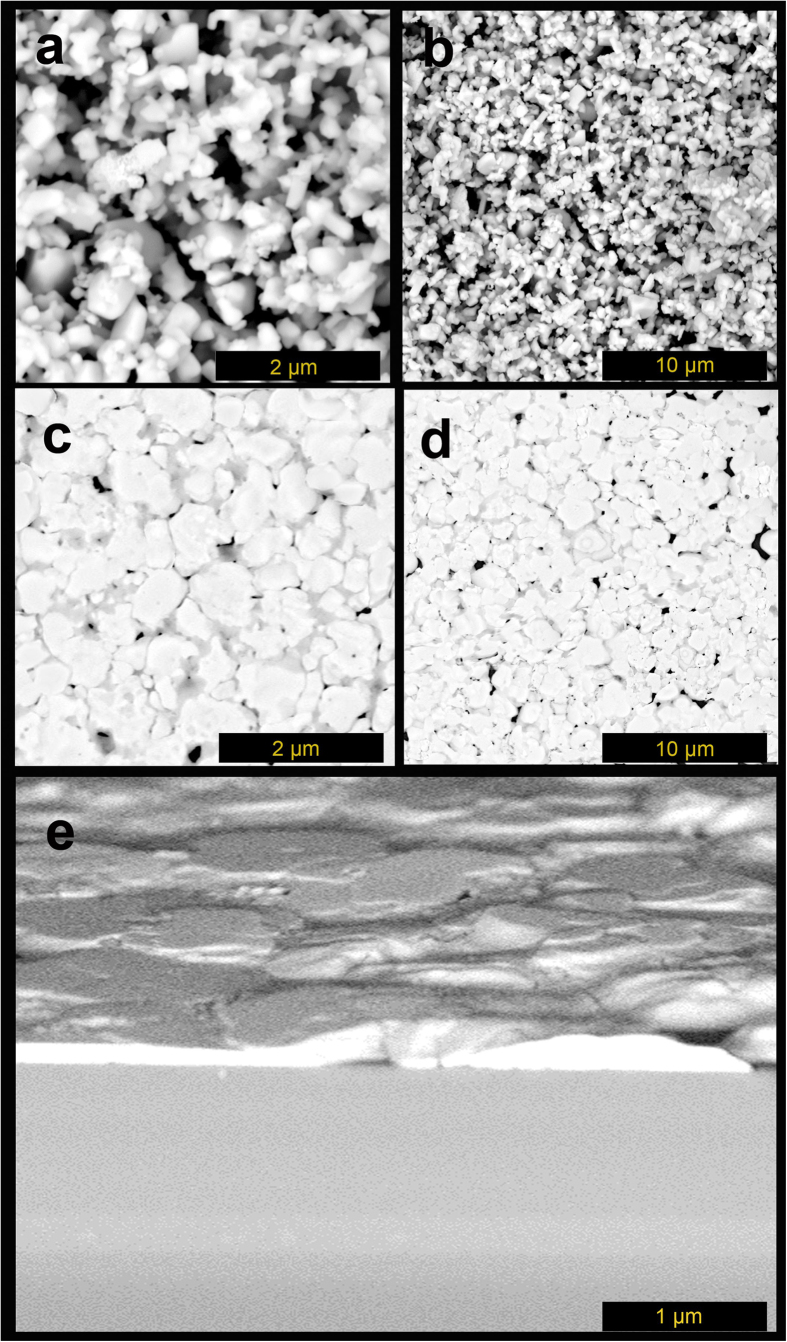
FE-SEM images of SFP layer by 20 spray passes (**a**,**b**); hot compressed SFP layer (**c**,**d**); and cross sectional images of compressed SFP layer (**e**).

As shown in Fig. 7a,b, the 20 passes of perovskite particles spraying provides good surface coverage by which the compressing step unifies the surface of the perovskite to an almost smooth and lower pinholed perovskite layer (Fig. 7c,d). Decreasing the thickness of perovskite layer by compressing step (Fig. 7e) declines the carriers recombination as shown in Fig. 5a 28.

### Compression pressure and time effects in device performance

**Figure 8:**
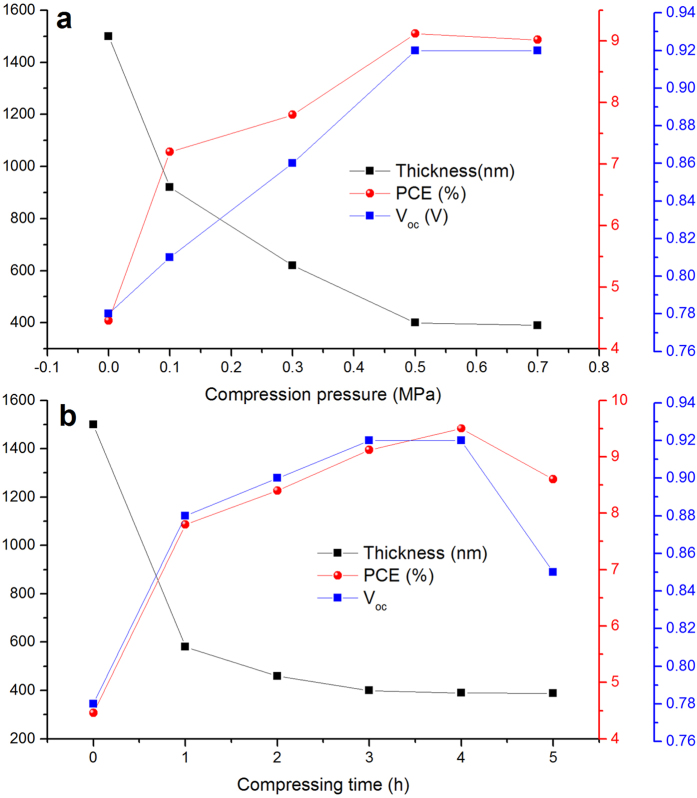
Compression pressures (**a**) and times (**b**) effect on perovskite thickness, PCE, and V_oc_ in preparation of SFP solar cells.

According to the fact that compression pressure and time are two important parameters in compressing the SFP layers, the effect of compression pressure and time on the perovskite layers were studied. As shown in the Fig. 8a, increasing the compression pressure up to 0.5 MPa decreases the thickness of as-sprayed SFP layer to 400 nm while further increase in compression pressure up to 0.7 MPa did not change the thickness of perovskite layer considerably. It is worthy to note that under higher compression pressure of 0.9 MPa, the quartz substrate could not tolerate the pressure because of its brittle characteristic. The PCE of the compressed perovskite layers efficiently raised by increasing the compression pressure which come from improving the perovskite uniformity causing lower charge recombination due to fewer pinholes in perovskite layer and better contacting of perovskite particles with substrate layer. To study the compression time effect on perovskite layer thickness and PCE, compressing the as-sprayed SFP layer under 0.5 MPa pressure for different times of 0 to 5 hours were investigated (Fig. 8b). Compressing the as-sprayed perovskite layer for 1 hour considerably decreased the perovskite thickness up to 580 nm showing 74% enhancement in PCE. Further compression up to 3 hours dramatically dropped the perovskite thickness to 400 nm showing 9.07% PCE. Compression the perovskite layer up to 4 hours did not affect the perovskite thickness and PCE of the devices. By further compression up to 5 hours, despite no considerable change in thickness of perovskite layer, the PCE and V_oc_ of the devices began to decrease probably due to higher generated defects in the interface of perovskite and TiO_2_ layers, which would, led to higher recombination. Hence, compression the as-sprayed SFP layer at 0.5 MPa for 3 hours would be the proposed optimized condition for solution-free perovskite solar cells.

### Photovoltaic performance in Inverted Architecture

**Figure 9:**
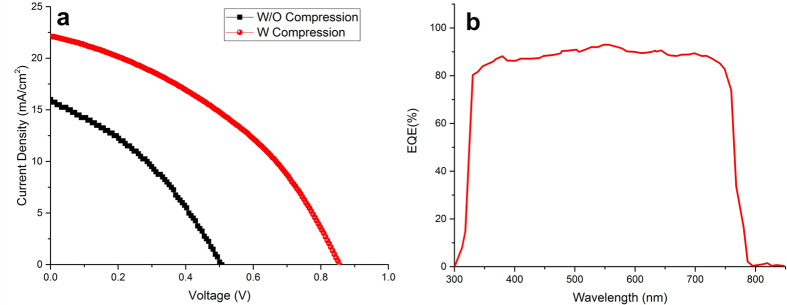
J-V curves measured at AM1.G solar illumination for devices prepared with inverted architecture of FTO/CuI/SFP/PCBM/Al before and after compression of SFP layer (**a**) and EQE curve of inverted compressed perovskite solar cell (**b**).

To evaluate deposition technique on sensitive layers, we deposited the modified 20-pass spray coating and compression of perovskite particles on the 40 nm cuprous iodide thin film to fabricate the inverted perovskite solar cell with the device structure of FTO/CuI/SFP/PCBM/Al. As shown in Table 2 and Fig. 9a. the champion respective PCEs of 2.80% and 7.71% were achieved for devices fabricated by SFP layer with and without compression process. The high current density of devices without compression shows low resistance of device layers while compressing the perovskite particles improves the interface charge extraction potential, which results in higher current density.

As widely reported, cuprous iodide shows lower resistance and high hole mobility in comparison with organic HTMs like spiro-OMeTAD^13^,^29^,^30^,^31^. The lower resistivity of cuprous iodide would potentially incline the current density of fabricated device. As observed in the present work, the devices fabricated by employing cuprous iodide and spiro-OMeTAD as the respective inorganic and organic HTMs with the same perovskite preparation method showed different current densities (Figs 5 and 9a). The higher achieved current density of devices prepared by cuprous iodide than the one prepared with spiro-OMeTAD can be attributed to the fast charge extraction potential, lower resistivity, and high hole mobility of CuI than spiro-OMeTAD layer^29^,^30^. As shown in Fig. 9a, compressing the perovskite particles on the cuprous iodide layer considerably increases the open circuit voltage and fill factor because of better surface coverage and the drop in a number of recombination sites in the interface of CuI and PCBM layers. The integrated current density of 21.8 mA/cm^2^ from incident photon to electron conversion efficiency (IPCE) analysis is in good agreement with the measured current density obtained from solar simulator (Fig. 9b).

### Hysteresis Study

**Figure 10:**
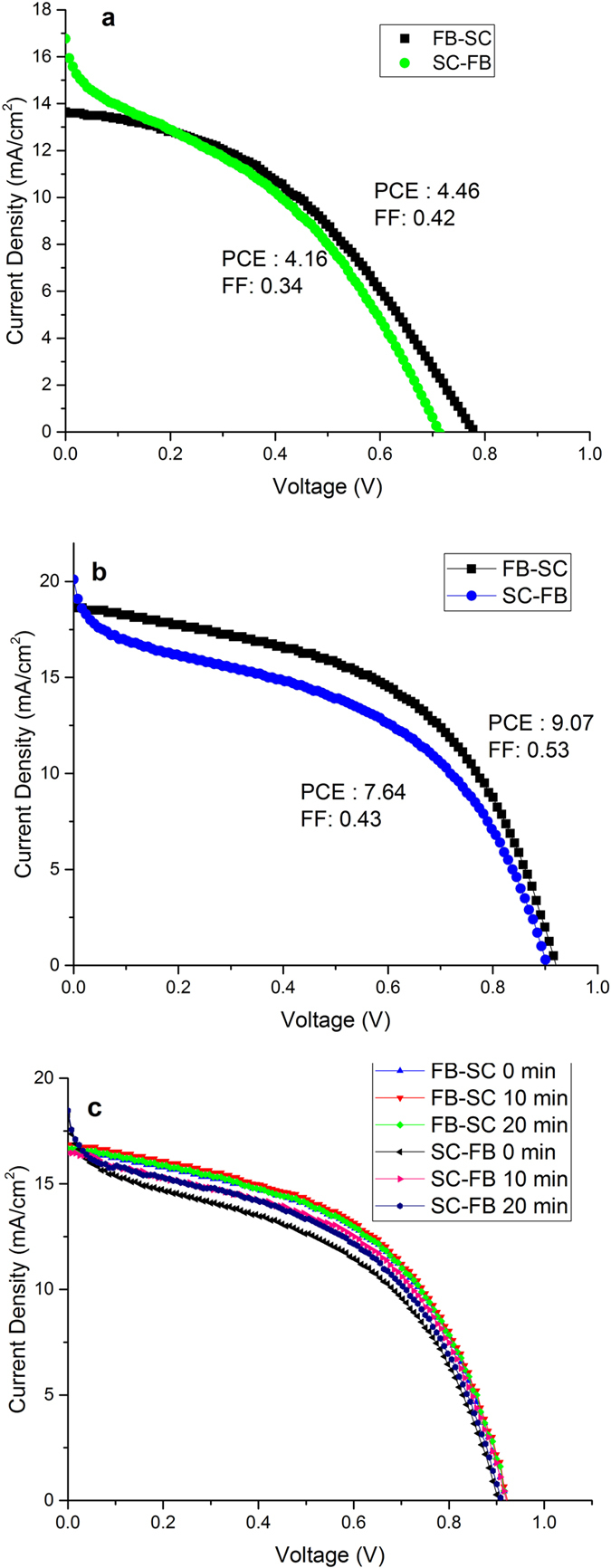
J-V curves of devices prepared with SFP before (**a**) and after (**b**) compression in normal devise structure of FTO/TiO_2_/SFP/spiro-OMeTAD/Au for the scan directions of forward bias to short circuit (FB-SC) and short circuit to forward bias (SC-FB), and effect of different post annealing times of 0, 10, and 20 minutes at 130 °C on device hysteresis (**c**).

To evaluate the hysteretic behavior of devices prepared by SFP in normal architecture, J-V curves were recorded every 10 mV at a scan rate of ~0.5 V s^−1^ in two scan directions, including forward bias to short circuit and short circuit to forward bias, for both devices fabricated by as-deposited perovskite particles by 20 spray coating passes and compressing perovskite particles (Fig. 10a,b). As shown in Fig. 10a,b, both devices show hysteresis that is possibly generated from the interface effect and unbalanced J_e_ and J_h_ in the perovskite solar cells^32^. Respective 6.7% and 14.2% difference in PCE of the devices in different scan directions was observed in devices prepared via as-spray deposited SFP and compressed perovskite particles (Fig. 10a,b). Despite higher efficiency of devices with compressed SFP layers, the observed hysteresis is larger in comparison to as-sprayed SFP layers. In the compressed PSCs despite the uniform perovskite layer and lower pinhole structure than as-sprayed SFP layer, the hysteresis is much considerable which may come from other reasons than pinholes. According to the fact that one of probable reason in the hysteresis effect is trapping and detrapping the transporting carriers, we studied the defects effect in the perovskite solar cells. Compressing is a mechanical treatment, which may cause too many structural defects in the compressed layers. Hence, declining the generated defects in the compressed perovskite layer can enhance the carrier transportation behavior. Heat treatment is one of well-known method in decreasing the defects. Accordingly, a set of heat treatment processes was designed to study the hysteresis of device. After hot pressing which was conducted at 0.5 MPa and 120 °C for 3 h, the compressed SFP layers were annealed at 130 °C in nitrogen atmosphere for 10 and 20 minutes under no compression pressure. As shown in the Fig. 10c, heat treatment of SFP layers for 10 minutes decreases the hysteresis of devices up to 48%. This means, under no pressing, by heat treatment of the layers, the generated defects in the perovskite structure and in the perovskite/TiO_2_ interface decreases, which results in declining the hysteresis in perovskite solar cells. Further annealing up to 20 minutes slightly decreases the cell efficiency in both backward and forward scan directions probably because of slight degradation of perovskite layer in the TiO_2_ and perovskite interface.

### Optical Study

**Figure 11:**
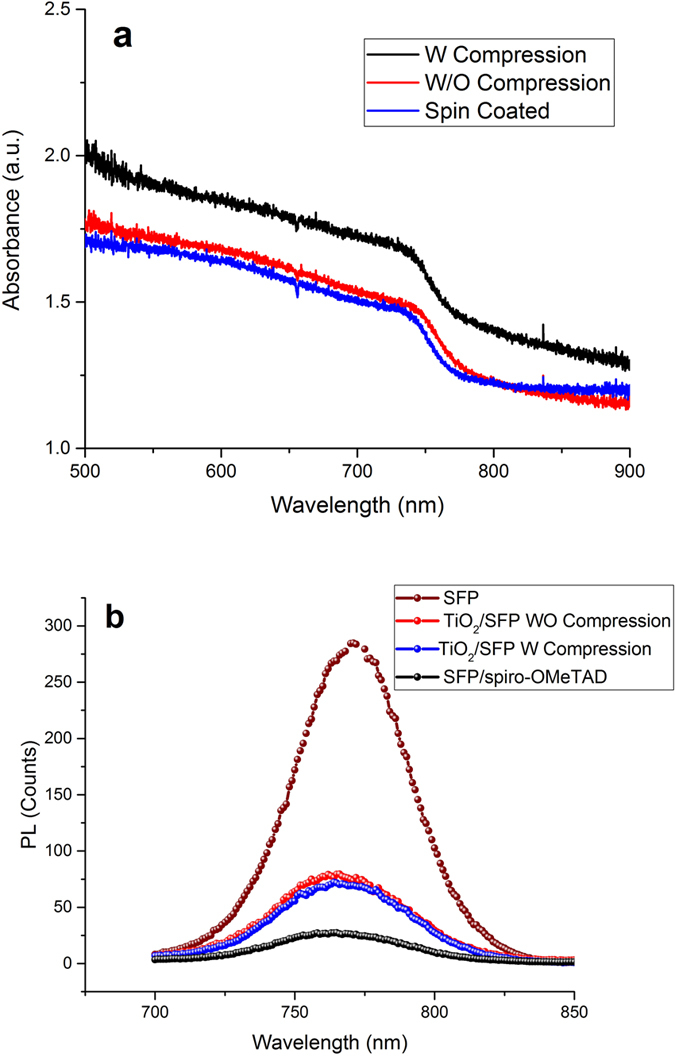
Absorbance spectra of SFP with and without compression compared to spin coated perovskite layer (**a**) and photoluminescence study of SFP with and without compression on glass, TiO_2_, and spiro-OMeTAD on SFP.

To investigate the optical properties of perovskite layer deposited by spray coating followed by hot pressing, UV-vis spectroscopy of deposited layers on the glass substrate was conducted and compared to the spin coated perovskite by 1:1 molar ratio of PbI_2_:MA in DMF. The perovskite prepared by both methods was adjusted to yield the same thickness of 400 nm. As shown in Fig. 11a, by compressing the inhomogeneous spray coated perovskite particles to a smoother and thinner perovskite layer, the absorption of perovskite layers increased because of the higher absorbed portion of light by compressed perovskite layer. As shown in the schematic of charge transferring and recombination states in Fig. 5d, by compression of perovskite particles and spreading on the substrate surface, which results in decreasing the thickness and increasing the grain size of perovskite layer, a higher portion of substrate will be covered by perovskite materials, leading to absorption of a higher portion of light. Hence, as depicted in Fig. 11a, the particle coverage would play a considerable role in increasing the light absorption in perovskite with thickness above 400 nm. The spin coating of the perovskite layer through the one-step deposition shows almost same optical properties but lower absorbance probably due to the different perovskite morphologies than two-step deposition of perovskite in the present work. Thus, using spray coating of perovskite particles followed by hot pressing would be an appropriate method in deposition of perovskite layer with better surface coverage as it is proposed as a key method in deposition of perovskite layer on the sensitive substrate such as CuI and Cu_2_O.

Perovskite layer on an inert substrate shows a strong luminescence at 773 nm, depicting a narrow band gap of 1.5 eV. Exciting the perovskite layer by a 600 nm wavelength showed no luminescence of glass and TiO_2_ at the luminescence range of perovskite layer. To study the photoluminescence behavior of the double layers of perovskite and TiO_2_, perovskite layer was deposited on the glass/TiO_2_ layer based on the described method. The laser beam was entered from the opposite side of quencher layer. As shown in Fig. 11b, TiO_2_ layers could quench the luminescence of perovskite at 773 nm by strong charge extracting ability. Compressing the perovskite layer by hot pressing enhances the quenching ability of TiO_2_ layers probably because of increasing the contact area between perovskite and TiO_2_ layer and improving the charge extraction ability of TiO_2_.

As reported in some works, increasing the contacting surface area between perovskite and charge extraction materials enhances the cell performance^12^, through which this two-step deposition and post-treatment of perovskite layer can be an appropriate method in the successful deposition of perovskite layer on the various substrates even the layers sensitive to perovskite solvents and solutions. The variation in the quenching the perovskite photoluminescence by TiO_2_ layer with and without compressing shows better charge transferability of compressed perovskite layer in delivering the electron to TiO_2_ in the interface of TiO_2_ and perovskite layer. Hence, in addition to charge extraction role, the perovskite solar cells enhancement through the compressing could be attributed to the better surface coverage by perovskite layer and declining the carrier recombination possibility in the interfaces of TiO_2_ and spiro-OMeTAD layers.

In summary, we presented a successful technique of SFP deposition for fabrication of perovskite solar cells. Through this method, the as-prepared perovskite particles by gas transformation followed by ball milling were sprayed on the TiO_2_ and CuI as the respective non-sensitive and sensitive electron and hole transport layers. The produced layers then were compressed to reach a better surface coverage and contacting to sublayer. Therefore, presence of no solvent in dissolution of the perovskite precursors removes the possibility of perovskite hydration or decomposition and opens a new approach to deposition of perovskite layers on the sensitive sublayers, which may be affected by perovskite solutions. Besides, this technique may receive much attention in fabrication of commercial perovskite solar cells.

## Methods

### Preparation substrate and compact layer

FTO-coated glass substrates were patterned by Zn powder and 2M HCl solution etching. The patterned FTO substrates were cleaned by soap-deionized water solution, followed by ultrasonication at 50 °C deionized water, ethanol, and isopropanol, and then subject to an O_3_/ultraviolet treatment for 20 min. After cleaning the substrates, a compact layer of TiO_2_ was created by spin coating a 0.15 M titanium diisopropoxide bis(acetylacetonate) (75 wt% in isopropanol, Aldrich) in 1-butanol (Aldrich) solution at 2000 rpm for 60 s, followed by heating at 125 °C. After cooling down the coated layers to room temperature, a 0.3 M titanium diisopropoxide bis(acetylacetonate) solution in 1-butanol was spin-coated on the substrate followed by heating at 125 °C for 30 minutes. The procedure was repeated twice to make a pinhole-free dense TiO_2_ film^33^.

### Synthesis of CH_3_NH_3_I

The CH_3_NH_3_I was synthesized by reacting 24 mL of CH_3_NH_2_ and 10 mL of HI in a 250 mL round-bottom flask at 0 °C for 2 h with stirring. The precipitate was collected using a rotary evaporator through the careful removal of the solvents at 50 °C. The as-obtained product was re-dissolved in 100 mL absolute ethanol and precipitated with the addition of 300 mL diethyl ether. After repeating this procedure for three times, the final CH_3_NH_3_I was collected and dried at 60 °C in a vacuum oven for 24 h.

### Synthesis the perovskite suspension

For synthesizing the perovskite particles, PbI particles were produced by reaction of Pb(NO_3_)_2_ and HI in DI water under vigorous stirring at room temperature. The produced PbI_2_ was dissolved in hot water and, after decanting, it was recrystallized to reach high purity lead iodide powder. This process was repeated twice. The produced PbI_2_ powder was dried at 150 °C under vacuum to remove almost all adsorbed and absorbed water molecules. For preparation of perovskite particles, 462 mg of produced dried PbI_2_ was instantly moved into closed vessel and 476 mg of CH_3_NH_3_I powder was added. The PbI_2_ and CH_3_NH_3_I mixture were heated at 150 °C and pure nitrogen atmosphere for 12 h. The dark perovskite powder was washed by anhydrous 2-propanol three times and transferred into 20 mL ball milling jar containing 5 mL of 5 mg/mL CH_3_NH_3_I in anhydrous 2-propanol. The jar was loaded with 12 stainless steel balls with 1 mm diameter followed by injecting moisture-free nitrogen to remove the moisture air (Amin Asia Ball Mill, Iran). The ball milling was conducted at 250 rpm for 6 h. The ball-milled perovskite powder was washed by anhydrous 2-propanol and re-dispersed in 10 mL 2-propanol for further use.

### Deposition of solution-free perovskite layer

For deposition of SFP layer, the perovskite suspension was diluted 5 times with 2-propanol and spray coated on the substrate at 80 °C. Here, the 10-second delay was chosen between every spraying pass for evaporation of 2-propanol and better adhesion of SFP on the substrate. The spray passes were performed 5 to 30 times. The spray coating was conducted at 2 mbar, 1 mL/min flow rate, 2 cm/sec spray head movement, 20 cm spray height from hot substrate, and moisture-free nitrogen gas as an inert carrier gas. The deposited SFP layers were hot pressed by polished Teflon sheet at 0.5 MPa and 120 °C for 3 h.

### Deposition of perovskite layer by spin coating

Deposition of perovskite layer on thin TiO_2_ compact layers was done at room temperature and air atmosphere with 20% moisture content. For the perovskite layer, a 1:1 ratio of PbI_2_:CH_3_NH_3_I in DMF was stirred at room temperature overnight and spin-coated on TiO_2_ compact layers at 3000 rpm for 30 secs, and then annealed at 100 °C for 5 minutes.

### Deposition of Spiro-OMeTAD

Immediately after cooling down the annealed perovskite layers to room temperature, the spiro-OMeTAD-based hole transporting layer (72 mg spiro-OMeTAD, 17.5 μl lithium-bis(trifluoromethanesulfonyl)imide (Li-TFSI) solution (520 mg Li-TFSI in 1 ml acetonitrile) and 26.6 mg 4-tert-butylpyridine all dissolved in 1 ml chlorobenzene) was deposited by spin-coating at 2000 rpm for 30 sec.

### Metal contact layer

By keeping the deposited spiro-OMeTAD in a desiccator for 12 h, the thin 100 nm gold contact was deposited on the spiro-OMeTAD by thin stainless steel shadow mask to create a 0.1 cm^2^ active area. Deposition of gold contact with a same thickness of 100 nm was carried out immediately after deposition of copper oxide layers.

### Preparation of inverted SFP solar cells

To prepare inverted perovskite solar cell using SFP layer, a 150 nm CuI was deposited on the cleaned and etched FTO layer by thermal deposition method at a low deposition rate of 0.5 Å/sec. The grown CuI thin film was deposited by 20 passes spray coating of SFP and hot pressed to reach a compressed SFP on the CuI layer. The SFP solar cell was completed by deposition of 200 nm PCBM (in dichlorobenzene, 2 wt%) followed by annealing at 100 for 45 min. The prepared devices were thermal deposited by 150 nm aluminum thin film through the 0.1 cm^2^ stainless steel shadows mask.

### Thin film and device characterization

To study the microstructure and morphology of the deposited films, field emission scanning electron microscopy (FE-SEM, S4160 Hitachi Japan) were used. The phase structure and crystal size of films were also investigated by X-ray diffraction (XRD, Philips Expert- MPD). XRD was performed in θ-2θ mode using Cu-Kα with a wavelength of 1.5439 Å radiation. The optical characteristics of deposited films were analyzed by UV–vis spectroscopy using the wavelength range of 190–900 nm. The photocurrent-voltage (I-V) characteristics of solar cells were measured under one sun (AM1.5G, 100 mW/cm^2^) illumination with a solar simulator (Sharif solar simulator). Steady-state PL measurements were acquired using a Varian Cary Eclipse (USA) fluorescence spectrometer.

## Additional Information

**How to cite this article**: Nejand, B. A. *et al*. Novel Solvent-free Perovskite Deposition in Fabrication of Normal and Inverted Architectures of Perovskite Solar Cells. *Sci. Rep.* **6**, 33649; doi: 10.1038/srep33649 (2016).

## Supplementary Material

Supplementary Information

## Figures and Tables

**Table 1:** Performance parameters of perovskite solar cells prepared by with and without compressing of SFP.

Device	Voc(V)	Jsc (mA/cm^2^)	FF	PCE(%)
FTO/TiO_2_/**SFP**/spiro-OMeTAD/Au	0.78	13.64	0.42	4.46
FTO/TiO_2_/**Compressed SFP**/spiro-OMeTAD/Au	0.92	18.62	0.53	9.07
FTO/TiO_2_/**Spin Coated PSK**/spiro-OMeTAD/Au	0.97	19.71	0.59	11.28

**Table 2:** Performance parameters of perovskite solar cells prepared by with and without compressing of SFP in the inverted structure of FTO/CuI/SFP/PCBM/Al.

Device	Voc (V)	Jsc (mA/cm^2^)	FF	PCE (%)
FTO/CuI/SFP/PCBM/Al	0.50	16.01	0.35	2.80
FTO/CuI/ Compressed SFP/PCBM/Al	0.85	22.13	0.41	7.71
