# Supplementary material for: Novel Solvent-free Perovskite Deposition in Fabrication of Normal and Inverted Architectures of Perovskite Solar Cells
Source: Sci Rep. 2016 Sep 19;6:33649. doi: 10.1038/srep33649 (PMC5027556; doi:10.1038/srep33649)
Supplement: Supplementary Information [file srep33649-s1.doc]

**Supporting Information:**

**Novel Solvent-free Perovskite Deposition in Fabrication of Normal and Inverted Architectures of Perovskite Solar Cells**

Bahram Abdollahi Nejand^1^, Saba Gharibzadeh^2^, Vahid Ahmadi^3^,*, H. Reza Shahverdi^1^

^1^ Nanomaterials Group, Dept. of Materials Engineering, Tarbiat Modares University, Tehran-Iran

^2^ Department of Physics, Tarbiat Modares University, Tehran, Iran

^3^ School of Electrical and Computer Engineering, Tarbiat Modares University, Tehran-Iran

**
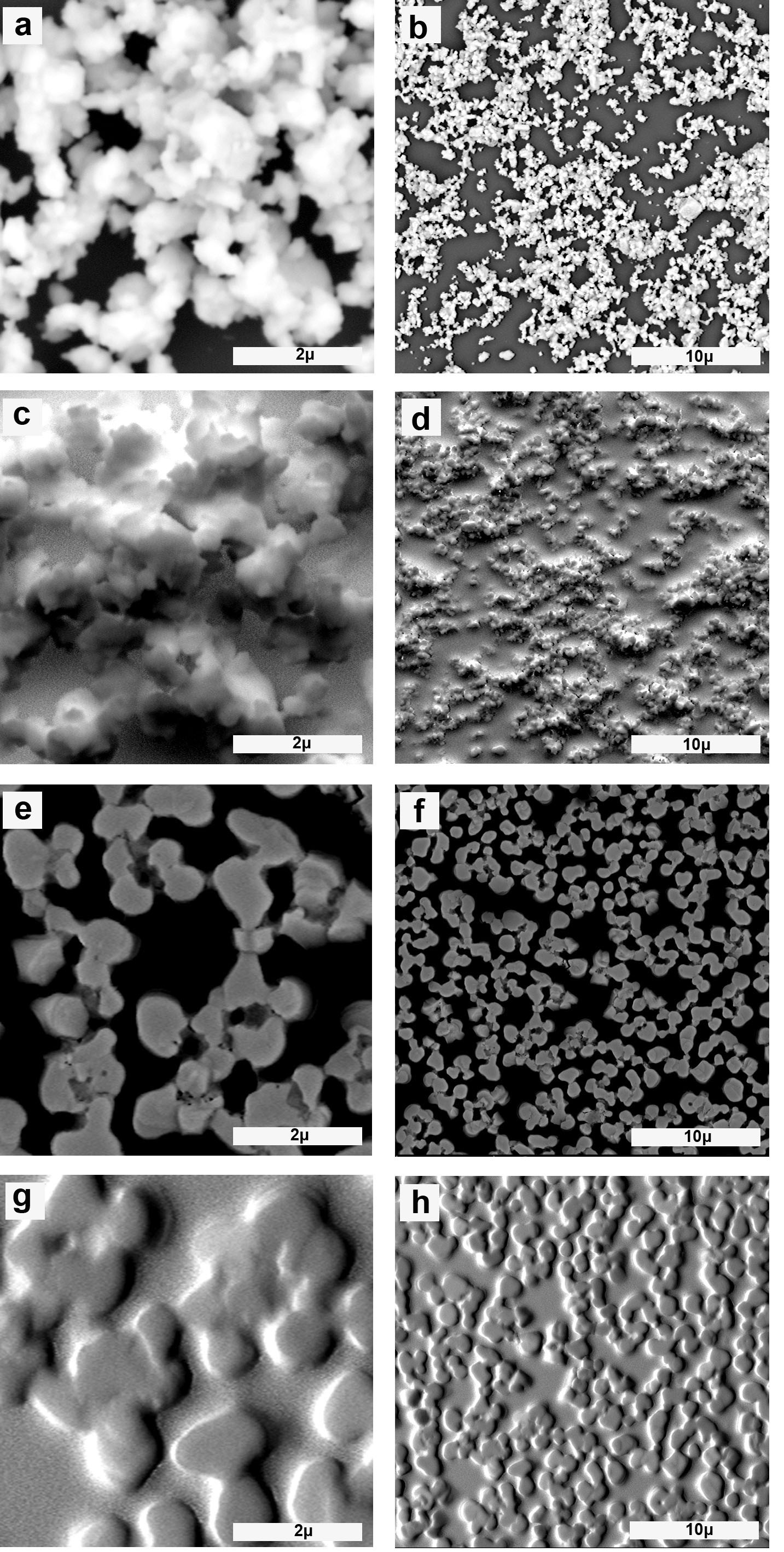
**

**Figure S1.** Normal (a, b, e, and f) and Topographic(c, d, g, and h) FE-SEM images of SFP layer by 10 spray passes (a, b, c, and d) and hot compressed SFP layer (e, f, g, and h).


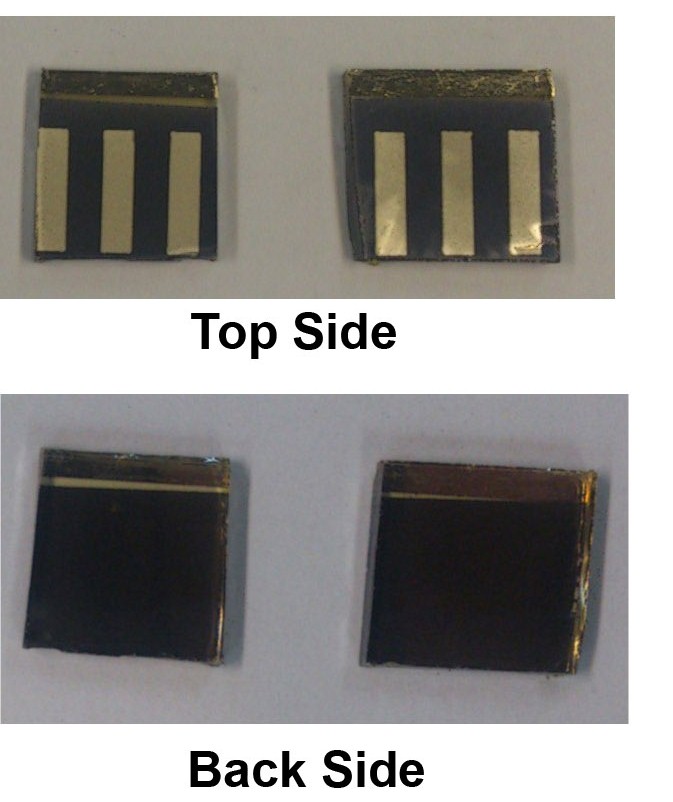


**Figure S2**.The photograph of top and back sides of FTO/TiO_2_/SFP/spiro-OMeTAD/Au


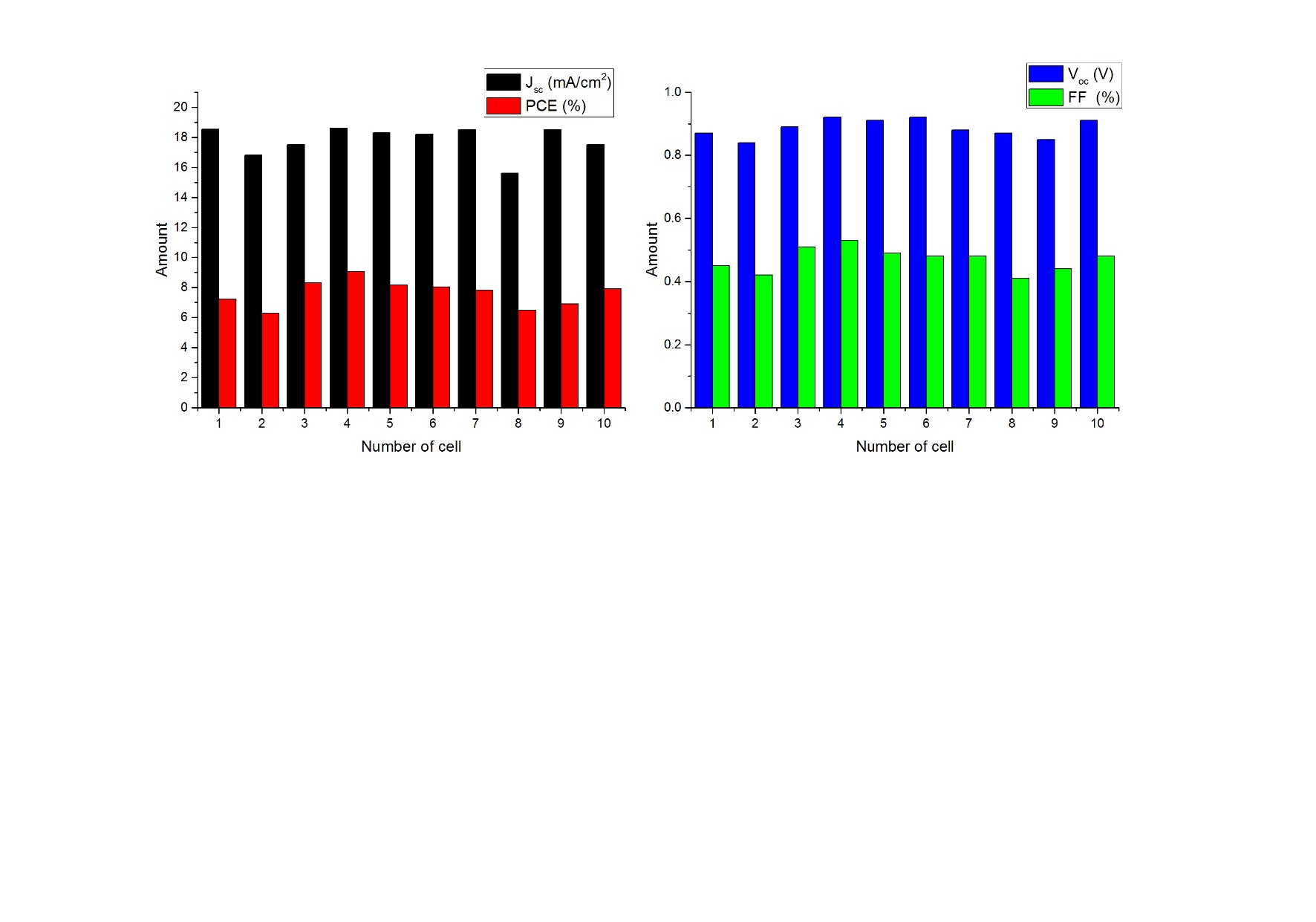


**Figure S3.** The J-V statistic data of 10 SFP solar cells.
